# Transverse Growth of the Maxillo-Mandibular Complex in Untreated Children: A Longitudinal Cone Beam Computed Tomography Study

**DOI:** 10.3390/s21196378

**Published:** 2021-09-24

**Authors:** Leah Yi, Hyeran Helen Jeon, Chenshuang Li, Normand Boucher, Chun-Hsi Chung

**Affiliations:** Department of Orthodontics, School of Dental Medicine, University of Pennsylvania, Philadelphia, PA 19104-6030, USA; leahyi@upenn.edu (L.Y.); hjeon@upenn.edu (H.H.J.); lichens@upenn.edu (C.L.); nsjboucher@gmail.com (N.B.)

**Keywords:** transverse growth, longitudinal study, CBCT, untreated

## Abstract

The aim of this study is to evaluate the longitudinal transverse growth of the maxillo-mandibular complex in untreated children using the Cone Beam Computed Tomography (CBCT). Two sets of scans on 12 males (mean 8.75 years at T1 and 11.52 years at T2) and 18 females (mean 9.09 years at T1 and 10.80 years at T2) were analyzed using Dolphin 3D imaging. The transverse widths of various maxillary and mandibular skeletal landmarks and the dentoalveolar and dental landmarks at the level of first molars were measured. Overall, there were greater increases in the transverse dimension in the posterior than anterior portions of the maxilla and mandible. The increase in intergonial width of the mandible seems to be primarily due to the lengthening of the mandibular body. The dentoalveolar process at the first molar level increases at an equal rate corono-apically and is independent to the changes in molar inclination. When comparing maxillary dentoalveolar changes with that of the mandible, greater increases were noticed in the maxilla, which might be explained by the presence of sutural growth in the maxilla. Moreover, the first molars maintain their coordination with each other despite the differential increase in the maxillary and mandibular dentoalveolar processes.

## 1. Introduction

Maxillary transverse deficiency is one of the most common skeletal deformities in the craniofacial region, leading to occlusal disharmony, facial asymmetry, and breathing problems [1,2,3]. Posterior crossbite, resulting from a narrow maxilla, is found in 7.7% of patients in the deciduous or mixed dentition, and its incidence increases into adulthood [4,5,6]. Additionally, many patients display dental compensation for skeletal transverse discrepancies, which could mean the incidence of a narrow maxilla is higher. Yet, the transverse dimension remains the least understood of the three dimensions of craniofacial growth. This is primarily due to a lack of reliable tools for an accurate analysis. Most of our current understanding of transverse growth of the maxillo-mandibular complex comes from longitudinal assessments of patients, who had implants inserted into their jaws and were evaluated with two dimensional posteroanterior (PA) and lateral cephalograms. Bjork and Skieller [7] found that, transversely, there is three times as much sutural growth between posterior implants in the infrazygomatic area than the anterior implants placed below the anterior nasal spine (ANS). Korn and Baumrind [8] found similar rates of transverse growth in the maxilla of about 0.4 mm/year and additionally used mandibular implants to characterize transverse growth of the mandibular dentoalveolar region at about 0.39 degrees per year.

Ricketts et al. [9,10] introduced the use of Jugale (J; the point of intersection of the maxillary tuberosity and zygoma) and Antegonion (Ag; the antegonial notch) as potential landmarks from which we could measure the transverse dimension of the maxilla and mandible, respectively. They reported that J-J increases by about 1 mm per year and Ag-Ag increases by 1.5 mm per year from age 3–21 years old, slightly more than their previous study that reported 0.6 mm/year and 1.4 mm/year respectively from age 9–16 [9,10].

Cortella et al. [11] reported similar initial rates of increase in J-J and Ag-Ag. However, contrary to Ricketts et al., they found that the rate is not sustained during growth and that it decelerates at different times depending on the gender. For females, J-J and Ag-Ag growths cease around 16 years old. For males, J-J only slightly reduces its growth rate, and Ag-Ag maintains its growth rate at least until 18 years old. Wagner and Chung [12] reported that the rate of transverse growth also differs among vertical facial types. J-J increases at a similar rate from age 6–14 years old for high angle, low angle, and normal females. However, Ag-Ag discontinues growth in high angle females earlier than that of low and normal angle females. The inconsistency among the literature reflects the limitations of PA cephalograms as a tool for evaluation. Two-dimensional radiography is distorted by radiographic enlargement, and the superimposition of structures makes it difficult to identify certain landmarks, as shown by Major et al. [13]. Head position adds another uncontrollable variable that influences measurements, as shown by Shokri et al. [14]. Furthermore, Lee et al. [15] investigated the alveolar and basal bone width in maxilla and mandible using CBCT and compared the maxillo-mandibular width values with conventional PA cephalograms including J-J and Ag-Ag. They found a statistically significant correlation in the maxillo-mandibular width between PA cephalograms and CBCT only at the first molar area, demonstrating the inaccuracy of transverse bony measurement using the PA cephalograms.

Moyers et al. [16] used dental models to demonstrate that intermolar width increases more in males than females from ages 6 to 16 years old and that maxillary intermolar width increases more than the mandibular intermolar width in both genders. While other studies using dental models found no gender differences in intermolar width, most agree with an initially increasing intermolar width that ceases with the development of full permanent dentition [17,18]. While dental models are a useful tool in assessing the transverse changes in the dentition, they are limited in an evaluation of transverse changes of the dentoalveolar and skeletal components of the face.

Through the advances in technology, CBCT has become a widely used form of diagnostic imaging in a clinical setting. CBCT eliminates errors from head positioning, magnification, and obstructed visibility. In addition, CBCT provides the high-resolution images with sub-millimeter voxel size (0.3–0.6 mm), short scanning times (5–9 s), and reduced radiation dose equivalent to 0.7–4.3 times that of a single standard panoramic radiograph (based on 3D Ceph setting in current CBCT) [19]. Three-dimensional imaging allows us to make transverse measurements in different coronal sections going antero-posteriorly, and hence more information can be gleaned from these images. For example, previous CBCT studies [20,21,22] have shown the presence of slight buccal and lingual inclination of the maxillary and mandibular 1st molars, respectively, and the uprighting of molars into adulthood. Along with other studies on expansion, Christie et al. [23] described the widening of the nasal cavity, midpalatal suture, and maxillary basal bone with expansion and that expansion occurs more inferiorly than superiorly.

CBCT studies also have the potential to create transverse norms, which we can use to diagnose “transverse discrepancies” and answer the question as to whether treatment of a perceived “transverse discrepancy” is necessary from the standpoint of keeping the dentition centered within the alveolar housing. Miner et al. [24] described a norm ratio of the width of the maxilla and mandible at the first molar and that those who fall under that norm tend to have posterior crossbites or excessive compensatory inclination of the first molars. While the potential of CBCTs in the evaluation of the transverse dimension is vast, the limitation in progress in the orthodontic field is the lack of images from untreated patients.

The purpose of this study is to evaluate the longitudinal transverse growth changes in untreated children using CBCT.

## 2. Materials and Methods

This study was approved by the University of Pennsylvania International review board and ethics committee (#830045). All CBCT images were oriented and analyzed using the Dolphin Imaging 3D software (version 11.9, Dolphin Imaging & Management Solutions, Chatsworth, CA, USA). We used the default setting, including the high-resolution and no sharpening under “General Options,” when we measured all CBCT images using the Dolphin software.

### 2.1. Sample

Thirty untreated subjects who had CBCT images taken at different periods were collected from various private practices utilizing CBCT imaging (i-CAT^TM^, KaVo Imaging, Hatfield, PA, USA) in routine records exams from November 2006 to July 2016. These CBCT images were taken at 120 kVp and 5 mA with the volume size of 16 × 13 cm^2^, a voxel size of 0.3 mm, and an exposure time of 3.7 s. The subjects were not treated at T1 for various reasons. For example, some patients were referred for ENT consultations due to hypertrophy of the adenoids and tonsils. Several sought second opinions and others postponed their treatment for financial reasons and came back for the treatment. The sample patients had skeletal Cl I or mild to moderate Cl II due to deficient mandible. Most patients had anterior arch perimeter deficiencies. Seven out of thirty patients had posterior crossbite (six buccal and one lingual crossbite). In addition, two patients had buccally impacted maxillary canine unilaterally, and two patients had congenitally missing maxillary lateral incisors bilaterally. The sample examined in this study includes 12 males who are an average age of 8.75 years old at T1 (5.4–11.48 yo) and 11.52 years old at T2 (8.7–14.7 yo) as well as 18 females who are an average age of 9.09 years old at T1 (6.2–11.7 yo) and 10.8 years old at T2 (7.2–13.7 yo).

### 2.2. Orientation

**Figure 1:**
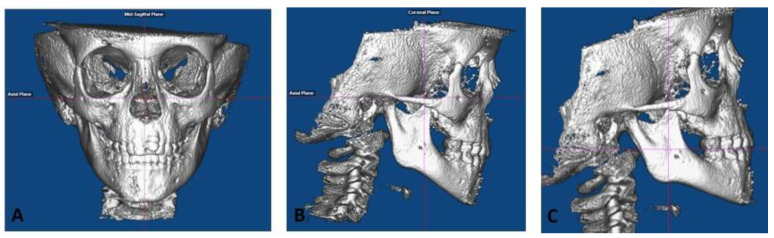
CBCT Orientation (**A**) Roll and Yaw, (**B**) Pitch (non-tooth-bearing, skeletal measurements), and (**C**) Pitch (tooth-bearing, dentoalveolar measurements).

- Roll: The cranium was oriented such that a midline could be drawn through the midpoint of the frontonasal suture and base of the nose and is parallel to the true vertical (Figure 1A). In addition, a true horizontal line goes through the most inferior aspect of the orbits.
- Yaw: The cranium was oriented to achieve the best symmetry of the cranium, zygomatic, and maxillary structures on either side of the midline (Figure 1A).
- Pitch: For measurements on the non-tooth-bearing, skeletal features of the face, the cranium was oriented such that the Frankfort horizontal plane was parallel to the true horizontal plane (Figure 1B). For measurements on the tooth-bearing, dentoalveolar areas, the cranium was oriented such that the functional occlusal plane was parallel to the true horizontal plane (Figure 1C).

### 2.3. Skeletal Measurements

**Figure 2:**
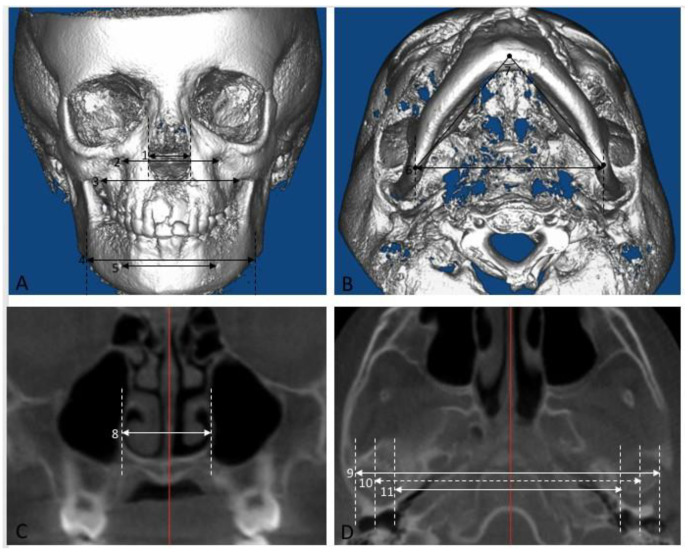
Skeletal measurements. The transverse width of various non-tooth-bearing areas of the maxilla and mandible was measured (“Skeletal measurements”). (**A**) 1. Anterior nasal width, 2. Io-Io, 3. Mx-Mx, 4. Ag-Ag, 5. Mf-Mf. (**B**) 6. Go-Go, 7. Gonion triangle. (**C**) 8. U6 nasal width. (**D**) 9. Cd- Cd lateral, 10. Cd-Cd middle, 11. Cd-Cd medial.

The transverse changes in the non-tooth-bearing, skeletal areas of the face were measured with the cranium oriented with Frankfort horizontal plane as the true horizontal plane. All measurements were parallel to the true horizontal plane. The following skeletal measurements were made as shown in Figure 2:

- Anterior nasal width: when viewed from the frontal, the distance between the most convex point along the lateral nasal margins.
- Io-Io: when viewed from the frontal, the distance between the mesiodistal and inferosuperior center of the infraorbital foramen.
- Mx-Mx: when viewed from the frontal, the distance between the most concave point in the infrazygomatic area.
- Ag-Ag: when viewed from the frontal, the distance between the most concave point in the lower mandibular border.
- Mf-Mf: when viewed from the frontal, the distance between the most mesial margin of the mental foramen.
- Go-Go: when viewed from the inferior, the distance between the mesiodistal center at the most infero-posterior point of the inferior mandibular border.
- Gonion triangle: when viewed from the inferior, the angle between the left and right gonion and menton. The points are identified on a profile view and the angle lies in the axial plane.
- U6 nasal width: the distance between the most convex point along the lateral nasal wall on a coronal section at the mesiodistal center of the maxillary 1st molar.
- Cd-Cd lateral: the distance between the most lateral points of the condyle that could be seen on axial cross sections of the condyle.
- Cd-Cd middle: the average of Cd-Cd medial and Cd-Cd lateral and represents the midpoint of the condylar head.
- Cd-Cd medial: the distance between the most medial points of the condyle that could be seen on axial cross sections of the condyle.

### 2.4. Dentoalveolar Measurements

The transverse width of the tooth-bearing areas on the T1 CBCT image were measured with the cranium oriented with the functional occlusal plane. The new true horizontal plane was transferred onto the T2 lateral cephalogram tracing on a maxillary superimposition as well as on a mandibular superimposition. The maxillary superimposition was done on the anterior surface of the zygoma, the maxillo-zygomatico-temporal sulci, and centered within the T2 tracing [7,25,26]. The mandibular superimposition was done using the “Structural Method [27,28]”. These transferred lines were used to orient the T2 CBCT image for the maxillary and mandibular dentoalveolar transverse measurements. This ensures that the T2 image was oriented in a way that would enable the observer to make measurements in areas comparable to that of the T1 image.

**Figure 3:**
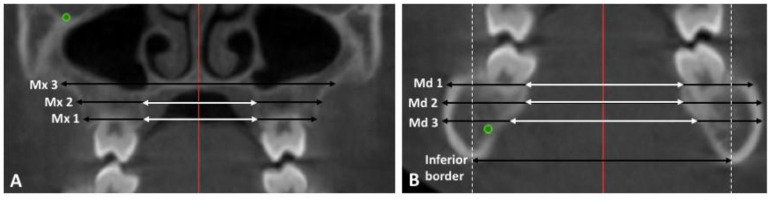
Dentoalveolar Measurements. (**A**) A coronal section at the mesiodistal midpoint of the maxillary first molar. The transverse width was measured at Mx 1, Mx 2, and Mx 3 at 2, 6, 10 mm apical to the U6 CEJ respectively. (**B**) A coronal section at the mesiodistal midpoint of the mandibular first molar. The transverse width was measured at Md 1, Md 2, Md 3 at 2, 6, 10 mm apical to the L6 CEJ respectively and inferior border. The black arrow shows the buccal transverse width, and the white arrow shows the lingual transverse width. The coronal and axial cross sections at which measurements were completed on the T2 image were identified by using the infraorbital and mental foramina.

On the T1 image, the transverse width was measured on axial cross sections at the following vertical levels as shown in Figure 3:

- Mx 1: 2 mm apical to the U6 CEJ on the T1 image. The most coronal axial cut on the T2 image.
- Mx 2: 6 mm apical to the U6 CEJ on the T1 image. The mid-axial cut between the most coronal and most apical cut on the T2 image
- Mx 3: 10 mm apical to the U6 CEJ on the T1 image. The most apical axial cut on the T2 image.
- Md 1: 2 mm apical to the L6 CEJ on the T1 image. The most coronal axial cut on the T2 image.
- Md 2: 6 mm apical to the L6 CEJ on the T1 image. The mid-axial cut between the most coronal and most apical cut on the T2 image
- Md 3: 10 mm apical to the L 6 CEJ on the T1 image. The most apical axial cut on the T2 image.
- Mandibular inferior border: the most inferior point on the mandibular border identified on a coronal section.

The buccal and lingual transverse widths were measured at the Mx/Md 1, 2, and 3 levels as described above on a coronal plane going through the mesiodistal center of the upper and lower first molars on the T1 image (U/L6) (Figure 3). On the T2 image, the location of this coronal plane and the Mx/Md 1, 2, and 3 levels were calculated and identified based on distance from the right infraorbital foramen for the maxillary measurements and the right mental foramen for the mandibular measurements. If the maxillary and mandibular superimpositions revealed the movement of Mf and Io, the x and y components of this movement were factored into the calculation.

### 2.5. Dental Measurements

**Figure 4:**
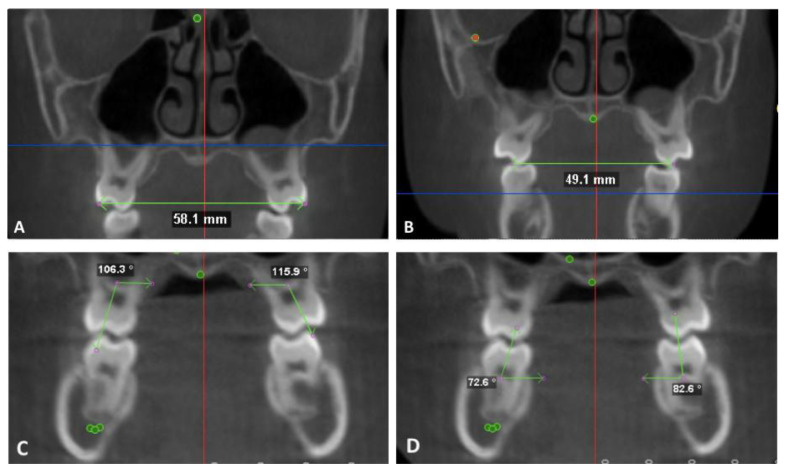
Dental measurements. Intermolar width and inclination with the maxillary and mandibular first molars. (**A**) maxillary intermolar width at the buccal cusp tips. (**B**) mandibular intermolar width at the buccal cusp tips. (**C**) maxillary molar buccolingual inclination (**D**) mandibular molar buccolingual inclination.

All dental measurements were made with the orientation by using the functional occlusal plane as the true horizontal plane of the T1 image. The intermolar measurements were made at the buccal cusp tips on the upper and lower first molar. The cusp tips were identified on coronal sections going through the midpoint of the buccal furcation of the right and left first molar (Figure 4A,B). The intermolar width is measured within a single coronal plane.

The molar inclination was measured as an angle formed by the long axis of the molar and true horizontal in a single coronal section. The long axis was identified by the mesiodistal and buccolingual center at the furcation and the deepest point of the occlusal table on the coronal section as previously described by Alkhatib and Chung [21]. The inner angle was measured for both maxillary and mandibular first molars (Figure 4C,D).

### 2.6. Statistical Analysis

The mean, standard deviations, and ranges were calculated for the resulting data showing the annual changes between the T1 and T2. A student paired T-test was used to confirm the statistical significance between T1 and T2 (*p* < 0.05). Intraexaminer reproducibility was tested by remeasuring 8 randomly selected patients at least 1 month apart (the same examiner, L.Y.). The intraexaminer error between two measurements was determined using a paired t-test. In addition, the measurement error was assessed by calculating the intraclass correlation coefficient (ICC). In addition, the Pearson correlation coefficient was measured to examine the strength between association of the two variables.

## 3. Results

### 3.1. Skeletal Measurements

**Figure 5:**
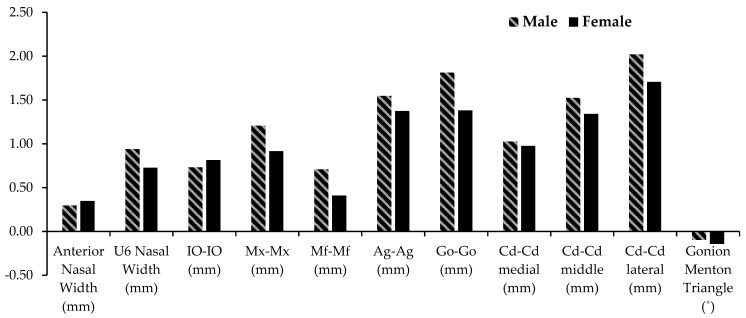
Skeletal transverse changes per year in the non-tooth-bearing regions of the maxilla and mandible for Males (stripes) and females (solid).

Table 1 and Figure 5 show the average annual changes for the transverse width of non-tooth-bearing skeletal structures of the face in males and females. All measurements except the gonion-menton triangle described the annual increases (*p* < 0.05). The gonion-menton triangle showed a slight annual decrease, but it is not statistically significant (*p* > 0.05). Among the maxillary and mandibular skeletal measurements, the anterior nasal width displays the smallest annual increases at 0.30 and 0.35 mm/year for males and females, respectively. The nasal width increases more posteriorly (U6 nasal width) at 0.94 and 0.73 mm/year for males and females, respectively. The intermaxillary width (Mx-Mx) has the largest annual increase of the maxillary transverse measurements at 1.21 and 0.92 mm/year in males and females, respectively. The inter-infraorbital foramen width (Io-Io) increases 0.73 and 0.81 mm/year in males and females, respectively. The inter-mental foramen width (Mf-Mf) increases 0.71 and 0.41 mm/year in males and females, respectively. Io-Io, Mx-Mx, and U6 nasal width increase significantly more than Mf-Mf in females only.

The inter-mental foramen width (Mf-Mf) has the smallest annual increases among the mandibular measurements and the second smallest overall in both genders. The inter-condylar width at the lateral aspect of the condyles (Cd-Cd lateral) increases at 2.02 and 1.71 mm/year in males and females, respectively. In males, the Cd-Cd lateral increases significantly more than all skeletal measurements except the inter-gonion width (Go-Go). In females, the Cd-Cd lateral increases significantly more than all but the Go-Go and inter-Antegonion width (Ag-Ag). The inter-gonion width (Go-Go) increases 1.81 and 1.38 mm/year in males and females, respectively. In both genders, the Go-Go increases significantly more than all maxillary measurements, Mf-Mf, and Cd-Cd medial. The inter-condylar width at the medial aspect of the condyles (Cd-Cd medial) increases 1.03 and 0.98 mm/year in males and females, respectively. The inter-condylar width from the middle of the condyles (Cd-Cd middle) is an average of the Cd-Cd lateral and Cd-Cd medial and is 1.52 and 1.34 mm/year for males and females, respectively. In males, the Cd-Cd med has significantly smaller increases than the Ag-Ag, Go-Go, Cd-Cd lat, and Cd-Cd mid. In females, the Cd-Cd medial has significantly smaller increases than the Go-Go and Cd-Cd lateral. The Cd-Cd middle has significantly smaller increases than Cd-Cd lateral in males only. The Cd-Cd middle and Cd-Cd lateral are larger than the Mf-Mf in both genders.

### 3.2. Dentoalveolar Measurements

**Figure 6:**
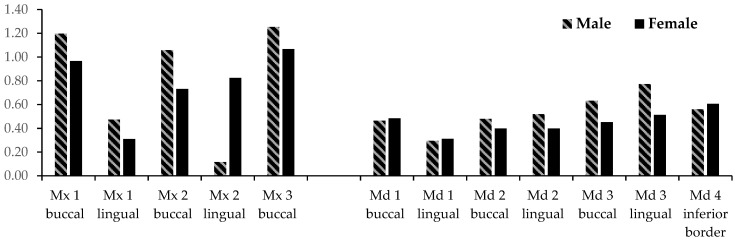
Dentoalveolar transverse changes per year at the first molar level of the maxilla and mandible for Males (stripes) and females (solid). Three coronal levels apical to the CEJ were examined. Mx/Md 1 is the most coronal level and Mx/Md 3 is the most apical level.

Table 2 and Figure 6 show the transverse changes in and near the tooth-bearing region in the maxilla and mandible, respectively. In the posterior aspect of the maxillary dentoalveolus at the 1st molar (U6), the buccal transverse width at the Mx 1, Mx 2, and Mx 3 levels increase at 1.20 mm/year, 1.06 mm/year, and 1.25 mm/year, respectively in males and 0.97 mm/year, 0.73 mm/year, and 0.82 mm/year, respectively in females. The transverse width, when measured from the lingual aspect of the alveolus at the Mx 1 and Mx 2 level, increases 0.47 mm/year and 0.12 mm/year, respectively in males and 0.31 mm/year and 0.82 mm/year, respectively in females. The lingual aspect of the alveolus could not be measured at the Mx 3 level because this level was superior to the palatal vault.

In the posterior aspect of the mandibular dentoalveolus at the 1st molar (L6), the buccal transverse width at the Md 1, Md 2, and Md 3 levels, and the inferior aspect of the mandibular border increases at 0.46 mm/year, 0.48 mm/year, 0.63 mm/year, and 0.56 mm/year respectively in males and 0.48 mm/year, 0.40 mm/year, 0.45 mm/year, and 0.61 mm/year in females respectively. The lingual transverse width at Md 1, Md 2, and Md 3 increases 0.30 mm/year, 0.52 mm/year, and 0.77 mm/year respectively in males and 0.31 mm/year, 0.40 mm/year, and 0.51 mm/year respectively in females.

The buccal transverse widths at Mx 1, 2, and 3 show greater annual increases than the lingual transverse width at Mx 1 in both genders. The differences among the buccal and lingual transverse widths at Md 1, Md 2, and Md 3 and the mandibular inferior border are not statistically significant in either gender. In males, the buccal transverse width at Mx 1, Mx 2, and Mx 3 have greater annual increases of all the mandibular dentoalveolar annual changes except the Md 3 lingual. In females, the buccal transverse width at Mx 1 and Mx 3 have greater annual increases than Md 1 buccal and lingual, Md 2 buccal and lingual, and Md 3 buccal.

### 3.3. Dental Measurements

**Figure 7:**
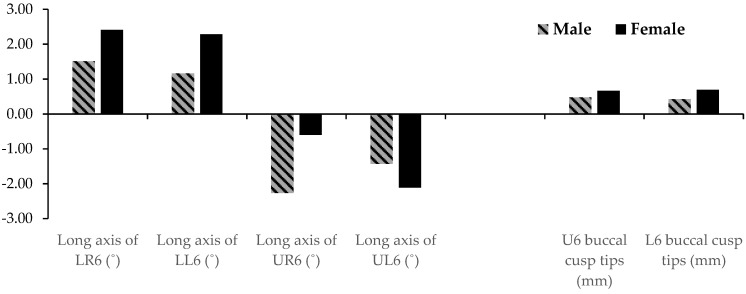
Dental transverse changes per year at the first molar level of the maxilla and mandible for Males (stripes) and females (solid).

Table 3 and Figure 7 show the annual transverse dental changes that occurred at the maxillary and mandibular first molars. The maxillary right and left first molars upright 2.27 degrees/year and 1.43 degrees/year respectively in males and 0.60 degrees/year and 2.11 degrees/year respectively in females. The mandibular right and left first molars upright 1.51 degrees/year and 1.16 degrees/year respectively in males and 2.41 degrees/year and 2.28 degrees/year respectively in females. The maxillary intermolar width measured at the cusp tips increases at 0.47 mm/year in males and 0.66 mm/year in females and the mandibular intermolar width at the cusp tips, which is 0.42 mm/year in males and 0.70 mm/year in females.

### 3.4. Intraexaminer Reliability

The intraexaminer reliability test revealed no statistically significant differences between the data collected for the re-analyzed sample of 8 random patients (*p* > 0.05). In each case, the ICCs ranged between 0.91 and 0.99, indicating excellent reliability.

## 4. Discussion

### 4.1. Skeletal

Our data portrays a trend in which the posterior maxillary and mandibular structures generally have greater increases in transverse width than the anterior structures in both genders. This is consistent with the findings of Bjork and Skieller [7], who attributed this observation to a greater opening in the midpalatal suture posteriorly than anteriorly. We found that the anterior nasal width increases less than posterior maxillary transverse measurements such as the inter-maxillary width (Mx-Mx) and the posterior nasal width (U6 nasal width) in both genders. Interestingly, annual increases in the Mx-Mx and the U6 posterior nasal width are positively correlated (R = 0.75, *p* < 0.01) in males, which is expected because Mx-Mx tends to be apical to the U6s. In the mandible, the same trend is observed. The inter-mental foramen width (Mf-Mf), which is the most anterior measurement on the mandible has smaller increases than most of the posterior mandibular transverse measurements, including the inter-condylar (Cd-Cd), inter-gonion (Go-Go), and inter- antegonion (Ag-Ag) widths in both genders. In general, males display higher average annual transverse increases than females, but this observation is only statistically significant for Go-Go. The lack of significance for the other measurements is likely due to the small sample size.

The inter- and intra-condylar width increases in both genders. The intra-condylar width increases as demonstrated by the greater increases in the Cd-Cd lateral than the Cd-Cd medial. Males have greater average increases in intra-condylar width (0.50 mm/condyle/year) than females (0.34 mm/condyle/year), but this difference is not statistically significant (*p* = 0.13), likely due to small sample size. The Cd-Cd middle represents the inter-condylar width at the middle of the condylar head and is the average of Cd-Cd medial and lateral. The Cd-Cd middle increases at about the same rate as the Go-Go and Ag-Ag in both genders, thus suggesting the posterior border of the mandible increases in a parallel fashion. In males, increases in Mx-Mx positively correlated with increases in the Cd-Cd middle (R = 0.61 and *p* = 0.04), and it increases at about the same rate as the Cd-Cd medial, suggesting that the width of the maxilla is coordinated with the posterior width of the mandible. The increasing inter- and intra-condylar width also suggests that the glenoid fossae also remodel and displace laterally to maintain their articulation with the condyles, in concert with the posterior maxilla. This is consistent with the findings of Ghoussoub et al. [21] and McLeod et al. [22], who found that the inter-glenoid and inter-condylar distance increases more in patients who were expanded compared to controls.

The minimal change in the gonion triangle suggests that the increase in the posterior mandibular width, including the Ag-Ag and Go-Go, is commensurate to the antero-posterior lengthening of the mandible that occurs with growth, and, thus, the posterior width of the mandible increases primarily due to the lengthening of the mandibular body and ramus. This is contrary to the findings of Gandini et al. [29] and Korn and Baumrind [8], who found an increase in the transverse width of the mandibular basal bone, which suggested a rotational increase in the mandible that is coordinated with the maxilla. The differences can be explained by the location of the implants, which were placed mid-body and on the buccal surfaces where surface apposition occurs from masticatory muscle insertion. McWade et al. [30] demonstrated through an examination of patients with a Frankel appliance that the amount of periosteal apposition in this region is affected by occlusal forces and cheek pressure, which are factors of masticatory muscle activity. Hence the mid- mandibular body area may show greater transverse increases than the more posterior Ag-Ag and Go-Go areas.

### 4.2. Dentoalveolar

The maxillary dentoalveolar transverse dimension increases primarily by opening of the midpalatal suture and secondarily by periosteal apposition [7,29,31]. The mandibular basal bone directly beneath the dentoalveolar process has been shown to increase its transverse dimension [8,29]. This likely occurs by periosteal apposition as the mid-symphyseal suture is fused at birth [31]. This would explain the generally greater increases in the buccal transverse width of the maxillary dentoalveolar process than that of the mandibular dentoalveolar process in both genders.

Not much is known about the transverse growth of the mandibular dentoalveolar region. This study demonstrates that the mandibular transverse width at the first molar increases at similar and small positive rates corono-apically from the crestal bone to the basal bone. The transverse width of the maxillary dentoalveolar process at the first molar also displays similar rates of increase corono-apically from Mx 1 to Mx 3 and Mx-Mx. It should be noted that Mx-Mx often coincided with the U6 area where the maxillary dentoalveolar measurements were done. It seems dentoalveolar development is independent of posterior dental inclination, which is shown to change with growth in this study.

The rate of increase in Ag-Ag is consistent with that of previous studies [9,10]. Ag-Ag and Go-Go, which represent the posterior mandible, increase statistically significantly more than all the mandibular dentoalveolar measurements (*p* < 0.05). This suggests that Ag-Ag cannot be used to predict the transverse growth of the mandibular dentoalveolar process and should be considered independent. This is in agreement with the study conducted by Hesby et al. [32], in which PA cephalometric x-rays were used to demonstrate that the transverse width of the mandibular dentoalveolar complex at the first molar seems to show only limited increases when compared to the transverse width at Go and Ag. This observation signifies the importance of understanding that the maxillary dentoalveolar process should be compared to the width of the mandibular dentoalveolar process rather than Ag-Ag because the mandibular dentoalveolar process does not have the same rate of growth as Ag-Ag.

The maxillary dentoalveolar process seems to increase in thickness by about 0.7 mm/year in both genders, which is indicated by the greater annual increase in Mx 1 buccal over that of Mx 1 lingual (*p* < 0.05). Further investigation would be needed to determine whether the increase in thickness is due to remodeling of the buccal and/or lingual aspect of the alveolus. The same pattern is not observed in the mandibular dentoalveolar measurements.

### 4.3. Dental

The maxillary and mandibular molars uprighted with age in agreement with Yang and Chung [20] and Alkhatib and Chung [21]. Both studies also reported greater uprighting of the mandibular molars than the maxillary molars to compensate for a greater transverse increase of the maxillary dentoalveolar process than the mandibular dentoalveolar process. In the female group of our study, only the LR6 uprights more than the UR6 (*p* < 0.05). The LL6 shows more average uprighting than the UL6, but this is not statistically significant (*p* > 0.05). In males, there was no statistically significant difference in lower versus upper molar uprighting, which may be due to the small sample size. Greater sample sizes could yield more information on differential molar uprighting in the maxilla and mandible as well as different bucco-lingual molar inclination among various facial types [33].

Despite the differential increases in the maxillary and mandibular dentoalveolar transverse widths, the maxillary intermolar width at the buccal cusp tips (U6 buccal cusp tips) increase at a similar rate as the mandibular intermolar width at the cusp tips (L6 buccal cusp tips), which would be expected as the maxillary and mandibular molars maintain a cusp to fossa relationship throughout growth.

### 4.4. Analysis Method

For the diagnosis of the transverse dimension of the maxillo-mandibular complex, several dental radiographic techniques are currently available, including PA cephalograms, Computed Tomography (CT), and CBCT. Optical Coherence Tomography (OCT) has been introduced in the medical field for noninvasive in vivo imaging such as ophthalmoscopy, angiography, and endoscopy [34,35,36,37]. Compared to past radiographic methods, these advances can provide better resolution with less exposure to radiation [38]. However, OCT has a much lower penetration depth of only 1 to 2 mm into the bone area. Therefore, for the orthodontic field, it has been used to examine the deformations and/or thickness of enamel [39,40,41,42,43,44,45].

The 3D CBCT has been considered the gold standard to examine the transverse skeletal and dental dimension with high sensitivity and specificity [24,46]. The landmarks and analysis methods used in our study have been tested or slightly modified from the previous publications, demonstrating a high degree of reproducibility, and confirming the statistical suitability of the methods [12,15,32,47,48,49,50,51,52]. For the skeletal measurements, J-J and Ag-Ag have been used to compare the maxillo-mandibular transverse discrepancy in previous publications. The use of CBCT can overcome several limitations resulting from PA cephalograms, such as distortion, overlapped structures, and magnification. Here, we added other measurements including the anterior nasal width and distance between the left and right infraorbital foramen/mental foramen/condyle for further evaluation. For the dentoalveolar measurements, we measured several points on both the buccal and lingual side corono-apically depending on the distance from CEJ to examine the differential growth. For the dental measurements, we used the intermolar width and molar inclination on the upper and lower first molars using CBCTs, which is more precise than the use of dental casts.

## 5. Conclusions

The following conclusions can be made from this study:

(1) The nasal cavity increases its transverse dimension more posteriorly than anteriorly in both genders.
(2) The transverse mandibular dimension increases more posteriorly than anteriorly in both genders.
(3) The intercondylar width increases at a similar rate to the posterior maxillary width (Mx-Mx), and the two are positively correlated.
(4) The inter-condylar width and inter-gonion width increase at the same rate. The posterior border of the ascending mandibular ramus grows laterally in a parallel fashion.
(5) The maxillary dentoalveolar process at the first molar generally has greater increases than the mandibular dentoalveolar process and mandibular inferior border at the first molar.
(6) The maxillary and mandibular dentoalveolar processes increase in width equally corono-apically. The growth of the alveoli seems to be independent of dental inclination.
(7) The maxillary and mandibular molars are upright with age, and the maxillary and mandibular intermolar widths, when measured from the buccal cusp tips, increase at an equal rate.

## Figures and Tables

**Table 1:** Skeletal Transverse Changes Per Year in Males and Females (non-tooth-bearing regions) (*, *p* < 0.05; NS, not significant).

		Male						Female					
		N	Mean	SD	Min	Max	*p*-Value	N	Mean	SD	Min	Max	*p*-Value
Maxilla	Anterior Nasal Width (mm)	12	0.30	0.23	−0.02	0.72	*	18	0.35	0.29	0.00	0.89	*
	U6 Nasal Width (mm)	10	0.94	0.40	0.58	1.87	*	17	0.73	0.27	0.23	1.14	*
	IO-IO (mm)	12	0.73	0.48	0.09	1.71	*	18	0.81	0.62	0.13	2.53	*
	Mx-Mx (mm)	12	1.21	1.04	0.03	4.03	*	17	0.92	0.71	0.00	2.62	*
	Mf-Mf (mm)	12	0.71	0.60	0.00	2.30	*	18	0.41	0.39	0.05	1.48	*
Mandible	Ag-Ag (mm)	11	1.55	0.59	0.43	2.30	*	17	1.37	0.84	0.13	3.33	*
	Go-Go (mm)	11	1.81	0.41	1.28	2.78	*	16	1.38	0.52	0.19	2.16	*
	Cd-Cd medial (mm)	12	1.03	0.58	0.35	2.59	*	17	0.98	0.52	0.08	1.85	*
	Cd-Cd middle (mm)	12	1.52	0.42	1.04	2.52	*	17	1.34	0.50	0.11	2.13	*
	Cd-Cd lateral (mm)	12	2.02	0.42	1.23	2.89	*	17	1.71	0.77	0.00	3.75	*
	Gonion Menton Triangle (°)	12	−0.10	0.44	−0.92	0.72	NS	17	−0.14	0.65	−1.23	0.82	NS

**Table 2:** Dentoalveolar Transverse Changes Per Year in Males and Females (*, *p* < 0.05).

		Male						Female					
		N	Mean	SD	Min	Max	*p*-Value	N	Mean	SD	Min	Max	*p*-Value
Maxilla	Mx 1 buccal	11	1.20	0.63	0.09	2.25	*	18	0.97	0.56	0.22	2.58	*
	Mx 1 lingual	11	0.47	0.55	−0.07	1.87	*	17	0.31	0.41	−0.24	1.22	*
	Mx 2 buccal	12	1.06	0.52	0.15	2.04	*	18	0.73	0.58	−0.36	1.56	*
	Mx 2 lingual	5	0.12	0.24	−0.09	0.53	*	8	0.82	0.64	0.00	1.95	*
	Mx 3 buccal	10	1.25	0.87	−0.07	2.87	*	17	1.07	1.06	−0.37	3.31	*
Mandible	Md 1 buccal	12	0.46	0.57	−0.42	1.22	*	18	0.48	0.93	−1.45	2.08	*
	Md 1 lingual	11	0.30	0.37	−0.37	0.93	*	18	0.31	0.49	−0.49	1.44	*
	Md 2 buccal	12	0.48	0.68	−0.22	1.74	*	18	0.40	0.74	−1.66	1.45	*
	Md 2 lingual	12	0.52	0.82	−0.28	2.52	*	18	0.40	0.62	−0.65	1.54	*
	Md 3 buccal	12	0.63	0.56	−0.25	1.62	*	18	0.45	0.76	−1.88	1.15	*
	Md 3 lingual	12	0.77	1.09	−1.01	2.45	*	18	0.51	1.00	−0.81	2.05	*
	inferior border	12	0.56	0.61	−0.02	2.09	*	18	0.61	0.55	−0.65	1.75	*

**Table 3:** Dental Transverse Changes Per Year in Males and Females (*, *p* < 0.05).

	Male						Female					
	N	Mean	SD	Min	Max	*p*-Value	N	Mean	SD	Min	Max	*p*-Value
Long axis of LR6 (°)	9	1.51	1.77	0.06	5.62	*	17	2.41	1.85	0.27	6.11	*
Long axis of LL6 (°)	9	1.16	1.23	0.15	3.57	*	17	2.28	2.03	−0.11	6.36	*
Long axis of UR6 (°)	9	−2.27	1.80	−6.05	−0.60	*	17	−0.60	1.06	−2.52	1.53	*
Long axis of UL6 (°)	9	−1.43	1.51	−4.43	−0.09	*	16	−2.11	1.43	−4.54	0.14	*
U6 buccal cusp tips (mm)	9	0.47	0.35	0.09	1.01	*	16	0.66	0.60	−0.19	1.88	*
L6 buccal cusp tips (mm)	9	0.42	0.44	−0.29	1.01	*	17	0.70	0.84	−0.34	2.88	*

## Data Availability

The data presented in this study are available on request.
